# Development of a new scoring method in the neurofunctional assessment of preterm infants

**DOI:** 10.1038/s41598-022-20754-y

**Published:** 2022-09-29

**Authors:** Odoardo Picciolini, Maria Lorella Giannì, Laura Messina, Nicola Pesenti, Monica Fumagalli, Laura Gardon, Chiara Squarza, Fabio Mosca, Camilla Fontana, Matteo Porro

**Affiliations:** 1 Fondazione IRCCS Ca’ Granda Ospedale Maggiore Policlinico, Pediatric Physical Medicine & Rehabilitation Unit, Milan, Italy; 2 University of Milan, Department of Clinical Sciences and Community Health, Milan, Italy; 3 Fondazione IRCCS Ca’ Granda Ospedale Maggiore Policlinico, NICU, Milan, Italy; 4 University of Milano-Bicocca, Division of Biostatistics, Epidemiology and Public Health, Department of Statistics and Quantitative Methods, Milan, Italy

**Keywords:** Paediatric research, Neonatal brain damage

## Abstract

Infants born preterm are at high risk of presenting neurodevelopmental delay. The Neurofunctional Assessment (NFA) describes infants’ neurodevelopment through the evaluation of six different domains. This study aimed to evaluate how, in a cohort of preterm infants, each NFA domain assessed at 3 months of corrected age (CA) was associated with neurodevelopment at 2 years of CA using the Griffiths Mental Developmental Scales Extended Revised (GMDS-ER). In addition, by introducing the NFA complexity score (CS), the study aimed to define a threshold that can help clinicians discriminate infants at higher risk of later neurodevelopmental delay. We conducted an observational, longitudinal study including 211 preterm infants. At 3 months of CA, infants who had normal scores in each domain showed a significantly higher GMDS-ER global quotient (GQ) at 2 years of CA. In addition, linear model results showed a significant negative relationship between the NFA CS and 2-year GMDS-ER GQ (estimate: − 0.27; 95% CI − 0.35, − 0.20; *p* value < 0.001). Each 10-point increase in the NFA CS was associated with an average 2.7-point decrease in the GMDS GQ. These results highlight how the NFA domains and NFA CS are compelling instruments for the early identification of children at risk for long-term adverse outcomes.

## Introduction

Preterm infants who are born with very low birth weight (VLBW) or at a gestational age less than 32 weeks have a higher risk of developing cognitive and motor dysfunction than their term-born peers. Major abnormalities include cerebral palsy, cognitive impairment, and visual and hearing impairments^1,2^. Additionally, minor disturbances such as clumsiness, development coordination disorders, learning disabilities or socioemotional difficulties have been reported^3,4^.

These problems are often diagnosed after the first year of life; however, it is well known that preterm infants may exhibit earlier signs of altered neurobehaviour, such as lower attention and regulation, higher excitability, poorer quality of movements, less habituation and orientation to stimuli, and a higher number of reflexes with nonoptimal responses^5–7^. In turn, these poorly organized behaviours may affect dyadic relationships and parental responsiveness, which is a key factor in the foundation of cognitive and social development^8^.

Neurological assessment in preterm infants should promptly identify these signs, allowing for early intervention, guiding clinicians in communicating the diagnosis, directing support to parents, referring children at risk of disability to rehabilitation services, and identifying problems and possible solutions for paediatricians.

Nevertheless, the close relationship among nutritional, respiratory and neurodevelopmental comorbidities in severe prematurity often makes it difficult to disentangle the interplay between proper neurological problems and the impact of other comorbidities on a child’s behaviour^9^.

The difficulty in defining a single and reliable method for the early assessment and diagnosis of neurological disorders is due to the developmental changes in neurological and behavioural performances that parallel brain maturation in the first years of life and the fact that the observed problems are not all identifiable by one specific neurological sign but rely on different emerging functions.

Among the most useful assessment tools for preterm infants, Hadders Algra^10^ proposes the following: the Amiel-Tison (ATNE), the Touwen (TINE), the Hammersmith Infant Neurological Examination (HINE), and the Neurofunctional Assessment (NFA) by Picciolini et al.

The NFA is a global tool that focuses on the development of normal functions in preterm infants and is based on the International Classification of Functioning, Disability and Health (ICF) approach^11^. The NFA explores six of the main domains of early infant development: regulation & adaptedness, neurosensory function, behavioural function, spontaneous motor repertoire, evoked motor repertoire, and accessory neurological signs. The maximum score, defined as the maximum value of the assessed items, reflects the most severe functional impairment.

Previous studies have described the reliability of the global NFA score to identify preterm infants at risk of delayed neurodevelopmental outcomes; however, no studies have focused on the different domains evaluated by the NFA that describe neurodevelopment in early infancy and their association with preterm infants long-term outcomes^12–14^.

The hypothesis of the present study was that the evaluation of each domain of the NFA could better describe motor, cognitive and socioemotional development at 2 years of corrected age in a cohort of VLBW infants. In addition, the present study, focusing on the contribution of each NFA item, aimed to identify a threshold that can help clinicians discriminate infants at higher risk of later neurodevelopmental delay.

## Methods

### Patients

This study was part of a larger project that investigated the evolution of neurological development in a cohort of preterm infants with a birth weight less than 1500 g admitted to the NICU, Fondazione IRCCS Ca’ Granda, Ospedale Maggiore Policlinico, from January 2014 to April 2017.

Results regarding neonatal general movements (GMs) trajectories and their associations with neurodevelopment at three months of corrected age (CA) in the same cohort have been already published^15^.

After hospital discharge, all the infants entered the standard follow-up program that included paediatric and neurofunctional evaluations and parental educational support.

The study was performed in accordance with the ethical standards in the 1964 Declaration of Helsinki and its later amendments. The study was approved by the Ethics Committee of Milano Area B (No 759; date of approval: 07/04/2015). Parental informed consent was obtained from both parents.

### Study design

We conducted an observational, longitudinal study. Infants were scheduled to be prospectively followed from three months of CA up to 2 years of CA. The NFA was performed at three months of CA by three senior physicians (OP, MP, LM) who were blinded to the infants’ neuroimaging findings and who had not been involved in the infants' intensive care. The NFA assessors were all certified for the administration of this specific evaluation and had more than 10 years of experience in administering the NFA for high-risk preterm infants.

The neurodevelopmental assessment was performed at 2 years of CA using the Griffiths Mental Development Scale – Extended Revised (GMDS ER) by 2 trained physicians (LG, CS) who were unaware of the infants’ clinical histories and NFA scores at three months of CA^16^.

The infant baseline characteristics were collected from hospital charts. The recorded data included sex, birth weight and gestational age (GA), the range of GA at birth, small for gestational age (SGA) according to Fenton's growth chart^17^, twin birth, mode of delivery, Apgar scores at 1 and 5 min, Clinical Risk Index for Babies (CRIB) scores^18^, duration of hospital stay and postmenstrual age at discharge. The following neonatal morbidities were considered: retinopathy of prematurity (ROP) ≥ 3°^19^, severe bronchopulmonary dysplasia (BPD)^20^, medical and surgical necrotizing enterocolitis (NEC)^21^, and sepsis, which was defined as increased plasma levels of c-reactive protein associated with a positive blood culture. Brain lesions were defined according to the combination of findings on both cranial ultrasound (cUS) and brain magnetic resonance imaging (MRI) performed according to the local clinical imaging protocol that included sequential cUS scans from birth up to term equivalent age (TEA). Conventional brain MRI was performed only once at TEA.

Severe brain lesions were defined as grade III intraventricular haemorrhage and/or parenchymal haemorrhagic venous infarction^22,23^ and/or posthaemorrhagic ventricular dilation and/or focal cerebellar haemorrhage and/or cystic periventricular leukomalacia and/or more than 6 punctate white matter lesions and/or brain malformations. Infants affected by genetic syndromes and/or major congenital malformations were excluded.

### Measures

#### Neurofunctional assessment at three months of CA

The NFA at three months of CA included a total of 29 items subdivided into 6 domains (regulation and adaptiveness, neurosensory function, behavioural function, spontaneous motor repertoire, evoked motor repertoire, and accessory neurobehavioural facilitators), which describe the infants’ emerging abilities. Furthermore, each domain comprises specific items that integrate the observation of a newborn. A neurofunctional score ranging from 0 to 4 was assigned to each item evaluated and was classified as follows: 0, normal function; 1, immaturity of function (without limitations); 2, moderate impairment of function (possible but limited); 3, severe impairment of function (only possible with the use of facilitators or assisted devices); and 4, function is not possible. Regarding the domain, the score was defined as the maximum score obtained in the items belonging to each domain, with the maximum score reflecting the most severe functional impairment. Similarly, the overall NFA score (OS) was assigned as the maximum score presented in at least one evaluated domain. According to our previous studies^12–15^, children were further classified into 2 groups for analysis: those with a normal NFA score (scores of 0–1) and those with an altered NFA score (scores of 2-4). A detailed description of the NFA domains at 3 months of CA is reported in Appendix 1.

#### NFA complexity score

Considering that the NFA OS is defined as the maximum score obtained for all items, it is often difficult to differentiate the complexity of infants with equal overall scores. For this reason, a new score, called the complexity score (CS), was calculated as the sum of the score obtained for each item included in the NFA. This score ranges from 0 (if the child receives a score of 0 for each of the items of the NFA) to 116 (if a child receives a score of 4 for all 29 items of the assessment).

#### Neurodevelopmental assessment at 2 years of CA

Neurodevelopmental outcomes at 2 years of CA were assessed using the validated Italian translation of the GMDS-ER^24^. This tool specifically investigates neurodevelopment in the locomotor, personal-social, hearing and language, eye and hand coordination and performance areas and provides separate subquotients, with a mean of 100 and a standard deviation of 16, for each of the investigated areas. A global quotient (GQ), with a mean of 100 and a standard deviation (SD) of 12, is then calculated. A score > 2 SD below the mean indicates severe impairment, and a score >1 SD below the mean indicates mild impairment.

### Statistical analysis

Descriptive statistics of the population are presented using the mean (standard deviation) or median (interquartile range) for continuous variables and number (percentage) for categorical variables.

One-way ANOVA and the Kruskal‒Wallis test were used to compare NFA neurofunctional scores for continuous variables with normal and nonnormal distributions, respectively. Fisher’s exact test was used to compare qualitative data.

The relationship between the NFA (OS and CS) scores and 2 year GMDS score was studied using one-way ANOVA and a general linear model. For GMDS scores, both the general quotient and the subquotients were considered. The Tukey HSD test or pairwise Mann‒Whitney U test with a false discovery rate multiple comparison controlling procedure was used for post-hoc analysis with ANOVA and the Kruskal‒Wallis test, respectively. The receiver operating characteristic (ROC) curve was used to assess the discriminatory power of the NFA CS score in identifying infants with adverse GMDS neurodevelopmental outcomes at 2 years of CA. Youden's index optimal curve cut-off was chosen.

All tests were considered two-tailed, and a *p* value less than 0.05 was considered significant. All analyses were performed using R V.4.0.0 (R Foundation for Statistical Computing, Vienna, Austria).

## Results

A total of 211 very low birth weight (VLBW) infants born in the Fondazione IRCCS Ca’ Granda Ospedale Maggiore Policlinico NICU were included in the study.

Among them, 10 infants died, and 4 were transferred to another hospital before discharge. In addition, 1 infant with a genetic syndrome was excluded from the study. Moreover, 6 infants were lost to follow-up at three months and 6 were lost to follow-up at 2 years of CA and were therefore excluded from the study.

A total of 184 infants were included in the analyses.

The overall baseline characteristics of the cohort are summarized in Table 1.

**Table 1:** Baseline characteristics of the population included in the study.

Demographic features	Overall (184)
Gestational age at birth (weeks), mean ± SD	29.2 (2.3)
Range of gestational age at birth (weeks)	23–35.3
Birth Weight (g), mean ± SD	1098.3 (268.8)
Male, n (%)	84 (45.7)
Twins, n (%)	87 (47.5)
Monochorionic twins, n (%)	39 (21.2)
Apgar score at 1’, median (range)	7.0 (6.0; 8.0)
Apgar score at 5’, median (range)	8.0 (8.0; 9.0)
Caesarean Section, n (%)	165 (90.2)
Small for Gestational Age, n (%)	35 (19.0)
Days of Hospitalization, median (IQR)	61.5 (47.0; 89.2)
Maternal Age, mean ± SD	34.6 (5.8)
Severe Brain Lesions, n (%)	15 (8.2)
Other Comorbidities, n (%) NEC (both medical and surgical), n (%) Sepsis, n (%) ROP ≥ 3°, n (%) Severe BPD, n (%)	49 (26.6) 6 (3.3) 37 (20.1) 16 (8.7) 19 (10.3)

*NEC* (necrotizing enterocolitis); *ROP* (retinopathy of prematurity); *BPD* (bronchopulmonary dysplasia).

### NFA OS at three months of CA

At three months of CA, 38 (21%) of the infants had a normal NFA OS (score=0), 73 (39%) had a mild NFA OS (score=1), 56 (31%) had a moderate NFA OS (score=2), and 17 (9%) showed an altered NFA OS (score=3). No children had a score of 4 on the NFA. The characteristics and short-term morbidities of infants according to the NFA at three months of CA are reported in Table 2.

**Table 2:** Baseline characteristics and short-term morbidities across NFA OSs.

Demographic features	0	1	2	3	p value
Count, n (%)	38 (20.7)	73 (39.7)	56 (30.4)	17 (9.2)	
Gestational age at birth (weeks), mean ± SD	30.0 (2.1)	29.4 (2.2)	29.1 (2.3)	26.9 (2.1)	< 0.001
Range of gestational age at birth (weeks)	26–34.3	24–34.3	24–35.3	23–30	
Birth Weight (g), mean ± SD	1150.4 (236.0)	1133.2 (248.7)	1084.1 (283.4)	878.5 (281.2)	0.002
Male, n (%)	12 (31.6)	29 (39.7)	34 (60.7)	9 (52.9)	0.023
Twins, n (%)	21 (55.3)	30 (41.1)	27 (48.2)	9 (56.2)	0.454
Monochorionic twins, n (%)	8 (21.1)	13 (17.8)	13 (23.2)	5 (29.4)	0.723
Apgar score at 1’, median (range)	7.0 (6.0; 8.0)	7.0 (6.0; 8.0)	6.0 (5.0; 8.0)	5.0 (4.0; 7.0)	0.01
Apgar score at 5’, median (range)	8.0 (8.0; 9.0)	8.0 (8.0; 9.0)	8.0 (8.0; 9.0)	7.0 (7.0; 8.0)	0.015
Caesarean Section, n (%)	34 (89.5)	68 (93.2)	47 (83.9)	16 (100.0)	0.175
Small for Gestational Age, n (%)	8 (21.1)	16 (21.9)	10 (17.9)	1 (5.9)	0.484
Days of Hospitalization, median (IQR)	53.0 (41; 61)	61.0 (44; 82)	69.0 (48; 107)	122.0 (67; 178)	< 0.001
Maternal Age, mean ± SD	34.9 (6.4)	35.3 (5.9)	33.6 (5.4)	34.1 (5.4)	0.222
Severe Brain Lesions, n (%)	1 (2.6)	2 (2.7)	3 (5.4)	9 (52.9)	< 0.001
Other Comorbidities, n (%)	5 (13.2)	15 (20.5)	16 (28.6)	13 (76.5)	< 0.001
NEC (both medical and surgical), n (%)	1 (2.6)	1 (1.4)	1 (1.8)	3 (17.6)	0.022
Sepsis, n (%)	4 (10.5)	13 (17.8)	11 (19.6)	9 (52.9)	0.007
ROP > 3°, n (%)	1 (2.6)	3 (4.1)	5 (8.9)	7 (41.2)	< 0.001
Severe BPD, n (%)	0 (0)	1 (1.4)	10 (17.9)	8 (47.1)	< 0.001

*NEC *(necrotizing enterocolitis);* ROP* (retinopathy of prematurity); *BPD* (bronchopulmonary dysplasia).

*P* values were computed using one-way ANOVA or the Kruskal‒Wallis test for continuous variables and Fisher’s exact test for categorical variables.

The impaired infants (i.e., scores of 2-3) weighed less and had a lower GA than infants who showed a normal NFA OS. Furthermore, impaired infants were more likely to be male, experience the most severe neonatal morbidities (severe ROP, NEC, BPD, and sepsis), develop severe brain damage and have longer hospital stays.

### Comparison between the NFA OS at three months of CA and the GMDS score at 2 years of CA

**Figure 1:**
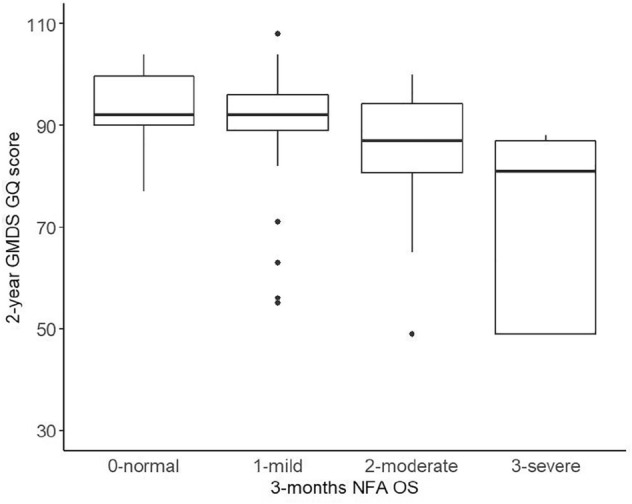
2-year GMDS global quotient score distribution according to NFA OSs at three months of CA.

The GQ was normal (88 or more) in 111 (60%) infants and 88 or lower in 73 (40%) infants. The 2 year GMDS GQ scores reflected the three-month NFA OS trend, with the highest global neurodevelopmental scores observed in infants with normal or mild NFA OSs; accordingly, the GMDS scores decreased significantly as the NFA scores increased (p value <0.001, one-way ANOVA). At post hoc analysis, all NFA groups had significantly different GMDS scores, except for the normal and mild NFA groups, which showed similar GMDS scores (*p* value=0.761; Tukey HSD post hoc test) (Fig. 1).

The significant trend observed for the general quotient was maintained for the GMDS subquotients with no differences observed between infants with normal and mild NFA OSs (Table 3).

**Table 3:** Global quotient and subquotient mean (SD) scores at 2 years of CA according to the NFA OS at three months of CA.

		GMDS						
		N	General Quotient	Locomotor	Personal Social	Hearing and Language	Eye and Hand Coordination	Performance
NFA	0-normal	38	93.6 (6.6)	97.4 (6.5)	93.0 (8.0)	91.8 (8.5)	95.9 (5.9)	93.9 (6.4)
	1-mild	73	91.6 (9.3)	97.2 (5.9)	89.9 (9.9)	89.8 (11.6)	93.9 (7.2)	94.1 (8.1)
	2-moderate	56	85.8 (10.9)	91.7 (11.0)	85.1 (10.9)	83.2 (13.5)	88.9 (10.7)	87.7 (10.7)
	3-severe	17	72.4 (16.6)	72.7 (19.6)	78.0 (13.3)	77.5 (17.8)	73.9 (16.9)	73.6 (17.4)

### Comparison between NFA domains at three months of CA and GMDS scores at 2 years of CA

According to our previous studies, when focusing on domains, we aggregated the normal and mild NFA scores, which were not significantly different, into the 0-1 group and compared the scores with those of subjects with moderate or severe impairment (2-3 group). Infants with scores of 0-1 in each domain at 3 months of CA had significantly higher GQ scores at 2 years of CA (Table 4).

**Table 4:** Mean (SD) GMDS global quotient scores at 2 years of CA according to the NFA domain scores at three months of CA. The p value refers to the t test. * number of 0–1; 2–3 subjects for each domain.

NFA domain	Subjects N.*	NFA score of 0–1	NFA score of 2–3	p value
Regulation & Adaptedness	137; 47	90.7 (9.6)	82.1 (14.9)	0.005
Neurosensory Function	158; 26	90.3 (10.3)	77.7 (14.5)	0.004
Behavioural Function	139; 45	91.1 (9.4)	80.4 (14.5)	0.001
Spontaneous Motor Repertoire	124; 60	91.4 (9.4)	82.4 (13.7)	< 0.001
Evoked Motor Repertoire	140; 44	91.1 (9.8)	80.2 (13.6)	< 0.001
Accessory Neurobehavioural Facilitators	129; 55	91.4 (9.3)	81.7 (14.0)	< 0.001

### Comparison between NFA CSs and GMDS scores at 2 years of CA

**Figure 2:**
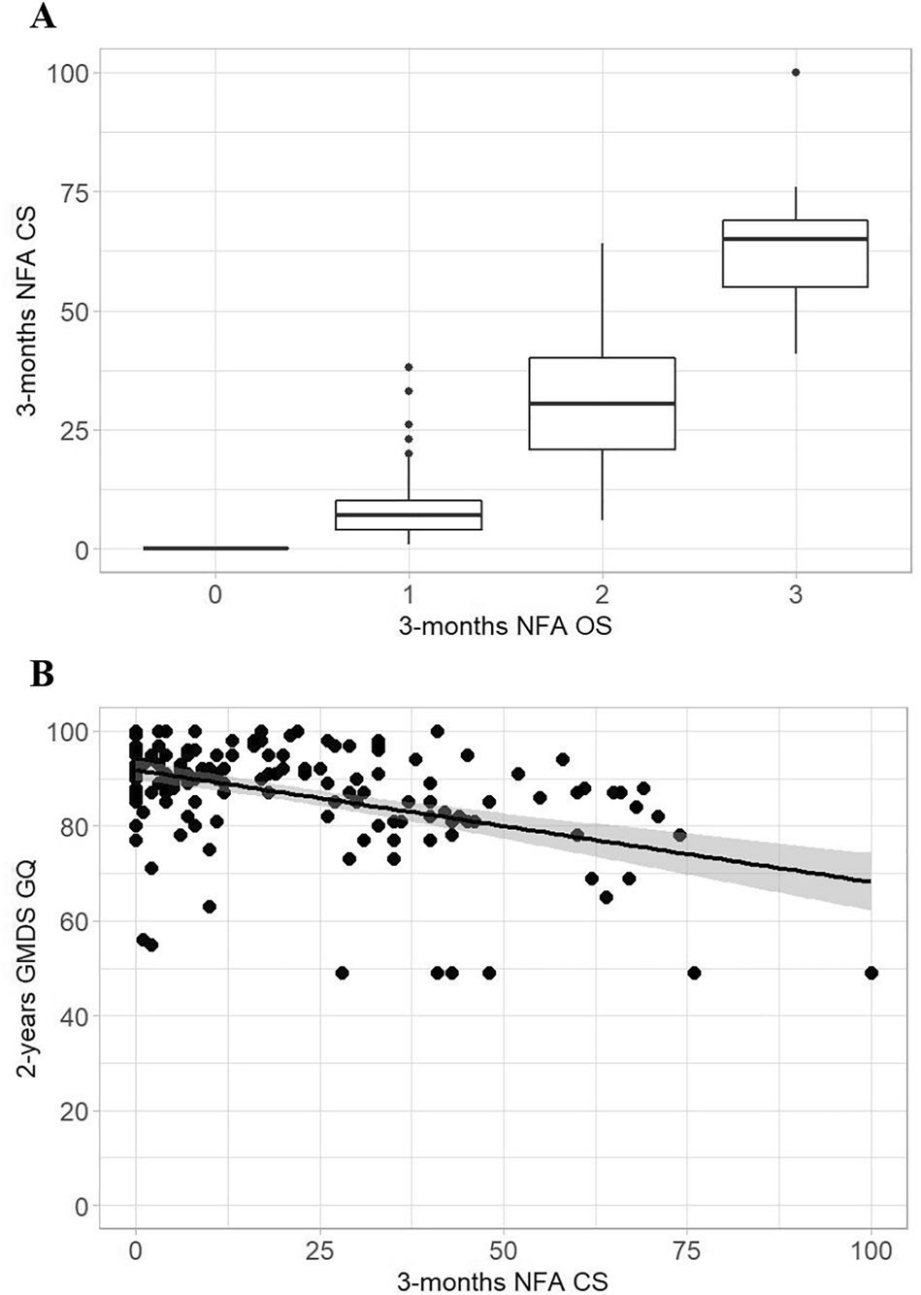
Panel (**A**) Distribution of NFA CSs at three months of CA according to NFA levels. Panel (**B**) Relationship between the NFA CS and GMDS GQ score at 2 years of CA. The black line represents the estimated linear model.

In our population, the NFA CS ranged from 0 to 100. Figure 2 Panel A shows the distribution of NFA CSs according to NFA evaluations. Linear model results showed a significant negative relationship between the NFA CS and 2-year GMDS GQ (estimate: −0.27; 95% CI −0.35, −0.20; *p* value<0.001) (Fig. 2 Panel B). Each 10-point increase in the NFA CS was associated with an average 2.7-point decrease in the GMDS GQ.

Using an NFA CS threshold of 26.5, the sensitivity and specificity of identifying infants with adverse neurodevelopmental outcomes at 2 years of CA (i.e., GMDS GQ < 88) were 0.89 and 0.62, respectively, with an AUC equal to 0.743.

## Discussion

This is the first study to investigate the relationship between different NFA domains at 3 months of CA and preterm infants’ neurodevelopment outcomes at 2 years of CA.

According to our previous study^14^, infants with an impaired global NFA profile (scores of 2–3) were more likely to exhibit a developmental delay at 2 years of CA, assessed by Griffiths scales considering both the GQ and subscales, than children who had a normal NFA profile at 3 months of CA.

In addition, infants who had a higher NFA OS at 3 months of CA also experienced a greater number of severe neonatal comorbidities, leading to a longer hospital stay. This is in line with previous studies^25^ showing how acute neonatal complications can affect normal neurodevelopmental trajectories, with infants with the most severe complications bearing a higher risk.

In the current study, when focusing on the NFA domains, infants with scores of 0-1 in each domain at 3 months of CA had significantly higher GQ scores at 2 years of CA.

Indeed, NFA domain items enable the simultaneous assessment of autonomic, behavioural, neurosensory and motor abilities, taking adaptability to the dynamic stimuli and the emerging functions into account, which in turn may influence long-term neurodevelopmental outcomes.

It is well known that early experiences have a crucial importance in establishing later neurodevelopmental processes^26^, and for preterm infants, this is particularly challenging due to the combination of the immaturity of the brain and premature exposure to the stressful environment in the NICU^27,28^. These factors can lead preterm infants to exhibit early alterations in sensory and behaviour modulation, as clearly depicted by the difficulties observed in our cohort in the 6 domains of the NFA at 3 months of CA.

In addition, our results highlight how these early difficulties may lead to negative neurodevelopmental outcomes. This is in line with previous studies^29^ showing how early behavioural signs can predict later neurodevelopment.

Furthermore, in this study, we introduced the NFA CS, which is calculated as the sum of the score reported for each item included in the NFA. The NFA CS allows for a more complete description of a child's development, capturing the relative role of each domain as a part of global development.

Our results showed a significant negative relationship between the NFA CS and the 2-year GMDS general quotient. Moreover, a threshold of 26.5 was also calculated to determine adverse outcomes at 2 years of CA.

The findings of our study are consistent with previous studies that found evidence of a clear association between neurodevelopment examinations and later outcomes, either at TEA or in the first months of CA^12–14^.

However, it must be noted that each assessment may differ considerably from others.

In particular, the ATNE, carried out at TEA, has good agreement with neurological and developmental assessments at later follow-up. Other studies suggest that the assessment of GMs, in combination with the HINE and MRI at TEA, represents the gold standard in the diagnosis of cerebral palsy in preterm infants^30,31^.

However, the increasingly frequent occurrence of developmental coordination disorders and other behavioural problems in premature babies has made it necessary to identify both motor and postural patterns and other features of infants’ development^32^. The Neonatal Behavioural Assessment Scale (NBAS), with its comprehensive evaluation, can highlight the progression of neurobehavioural performances and an infant’s adaptation to the surrounding environment, and it is a good predictor of subsequent behavioural problems in VLBW premature babies^33^. The NFA was developed as a global tool to give a comprehensive overview of infants’ neurodevelopment. In addition to the observation of infant neurobehaviour, along with postural and temporal patterns and adaptive and sensorineural functions, the NFA improved the understanding of various functional and developmental characteristics.

Moreover, the NFA is an additional method for assessing neurodevelopment in high-risk children; the functional approach in paediatrics and neonatology is also recommended by the WHO, as this strategy improves attention to the many aspects involved at the level of structures and functions (in line with the ICF-CY), allowing a more targeted follow-up^11,34^.

Recently, the NFA approach has been implemented in follow-up programs for preterm infants. In the Neuroprem study, neurofunctional subscale scores were significantly correlated with BSDI III composite scores and GMDS-R subscale scores, allowing the identification of all patients with cerebral palsy or other functional disabilities, leading to targeted intervention^35,36^.

The present study has some strengths and limitations. Relative to the methodology, one of the advantages is that infants were prospectively followed from 3 months of CA up to 2 years of CA, providing the opportunity to evaluate how the behavioural characteristics observed in the early period could affect long-term development. In addition, the present study analysed a large sample of VLBW infants, which strengthens the evidence of an integration between behavioural, cognitive and motor functions in preterm infant neurodevelopment and allows us to deepen the knowledge about the developmental features of preterm babies, which is essential to develop early intervention programs with a family-centred care approach.

Our study had some limitations. First, we collected data from one centre. Furthermore, our population was characterized by a lower percentage of males and a lower percentage of infants with severe BPD compared to other studies^37–39^, limiting the possibility of generalizing our findings from a single study centre to a large cohort of preterm infants.

Future studies are needed to confirm our interpretations and to have a full understanding of the NFA as a tool to assess preterm infant neurodevelopment.

## Conclusion

In conclusion, our study suggests that in a cohort of premature infants, the NFA and its domains, evaluated at three months of CA, can detect neurodevelopmental difficulties that infants will develop at 2 years of CA early.

These findings have important implications for clinical services and follow-up programs, as the provision of timely interventions depends on the accurate and early identification of children at risk for long-term adverse outcomes. Our findings support the timely activation of intervention programs so that early impairments at three months of age do not lead to greater neurodevelopmental difficulties at a later age.

## Supplementary Information

Supplementary Information.

## Data Availability

The data that support the findings of this study are available from the corresponding author upon reasonable request.

## References

[1] Moore T (2012). Neurological and developmental outcome in extremely preterm children born in England in 1995 and 2006: The EPICure studies. BMJ.

[2] Aarnoudse-Moens CSH, Weisglas-Kuperus N, van Goudoever JB, Oosterlaan J (2009). Meta-analysis of neurobehavioral outcomes in very preterm and/or very low birth weight children. Pediatrics.

[3] Marret S (2013). Brain injury in very preterm children and neurosensory and cognitive disabilities during childhood: The EPIPAGE cohort study. PLoS ONE.

[4] Wright LL (2006). What will it take to improve very low birth weight follow-up care?. Pediatrics.

[5] Spittle AJ (2016). Neurobehaviour and neurological development in the first month after birth for infants born between 32–42 weeks’ gestation. Early Hum. Dev..

[6] Brown NC, Doyle LW, Bear MJ, Inder TE (2006). Alterations in neurobehavior at term reflect differing perinatal exposures in very preterm infants. Pediatrics.

[7] Pineda RG (2013). Patterns of altered neurobehavior in preterm infants within the neonatal intensive care unit. J. Pediatr..

[8] Bornstein M, Tamis-LeMonda C (2001). Mother-Infant Interaction.

[9] Msall ME, Tremont MR (2002). Measuring functional outcomes after prematurity: developmental impact of very low birth weight and extremely low birth weight status on childhood disability. Ment. Retard. Dev. Disabil. Res. Rev..

[10] Hadders-Algra M, Heineman KR, Bos AF, Middelburg KJ (2010). The assessment of minor neurological dysfunction in infancy using the Touwen infant neurological examination: Strengths and limitations. Dev. Med. Child Neurol..

[11] Fontana C (2016). A longitudinal ICF-CY-based evaluation of functioning and disability of children born with very low birth weight. Int. J. Rehabil. Res..

[12] Picciolini O, Gianni ML, Vegni C, Fumagalli M, Mosca F (2006). Usefulness of an early neurofunctional assessment in predicting neurodevelopmental outcome in very low birthweight infants. Arch. Dis. Child. Fetal Neonatal Ed..

[13] Giannì ML (2007). Twelve-month neurofunctional assessment and cognitive performance at 36 months of age in extremely low birth weight infants. Pediatrics.

[14] Picciolini O, Montirosso R, Porro M, Giannì ML, Mosca F (2016). Neurofunctional assessment at term equivalent age can predict 3-year neurodevelopmental outcomes in very low birth weight infants. Acta Paediatr. Int. J. Paediatr..

[15] Porro M (2020). Early detection of general movements trajectories in very low birth weight infants. Sci. Rep..

[16] Battaglia, F. & Savoini, M. *GMDS-R Griffiths Mental Development Scales Revised 0–2 anni*. (Giunti OS, 2007).

[17] Fenton T (2003). A new growth chart for preterm babies: Babson and Benda’s chart updated with recent data and a new format. BMC Pediatr..

[18] Parry G, Tucker J, Tarnow-Mordi W (2003). CRIB II: An update of the clinical risk index for babies score. Lancet.

[19] International Committee for the Classification of Retinopathy of Prematurity. The International Classification of Retinopathy of Prematurity Revisited - An International Committ. *Arch Ophthalmol*. **123**, 991–999 (2005). 10.1001/archopht.123.7.991 16009843

[20] Jobe AH, Bancalari E (2001). NICHD/NHLBI/ORD workshop summary. Am. J. Respir. Crit. Care Med..

[21] Bell MJ (1978). Neonatal necrotizing enterocolitis. Therapeutic decisions based upon clinical staging. Ann. Surg..

[22] Parodi A (2020). Cranial ultrasound findings in preterm germinal matrix haemorrhage, sequelae and outcome. Pediatr. Res..

[23] Volpe, J. J. *Neurology of the Newborn* 5th edn, in 517–588 (Saunders Elsevier, Philadelphia, PA, 2008).

[24] Griffiths, R. & Huntley, M. *The Griffiths mental development scales-revised manual: From birth to 2 years*. (High Wycombe ARICD, 1996).

[25] Badr L, Bookheimer S, Purdy I, Deeb M (2009). Predictors of neurodevelopmental outcome for preterm infants with brain injury: MRI, medical and environmental factors. Early Hum. Dev..

[26] Tierney AL, Nelson CA (2009). Brain development and the role of experience in the early years. Zero Three.

[27] Ranger M, Grunau RE (2014). Early repetitive pain in preterm infants in relation to the developing brain. Pain Manag..

[28] Brummelte S (2012). Procedural pain and brain development in premature newborns. Ann. Neurol..

[29] Eeles AL (2013). Sensory profiles obtained from parental reports correlate with independent assessments of development in very preterm children at 2years of age. Early Hum. Dev..

[30] Ferrari F (2019). Motor and postural patterns concomitant with general movements are associated with cerebral palsy at term and fidgety age in preterm infants. J. Clin. Med..

[31] Noble Y, Boyd R (2012). Neonatal assessments for the preterm infant up to 4 months corrected age: A systematic review. Dev. Med. Child Neurol..

[32] Spittle A, Treyvaud K (2016). The role of early developmental intervention to influence neurobehavioral outcomes of children born preterm. Semin. Perinatol..

[33] Malak R (2021). Application of the neonatal behavioral assessment scale to evaluate the neurobehavior of preterm neonates. Brain Sci..

[34] Giovannetti AM (2013). Usefulness of ICF-CY to define functioning and disability in very low birth weight children: A retrospective study. Early Hum. Dev..

[35] Lugli L (2020). Neuroprem: The neuro-developmental outcome of very low birth weight infants in an Italian region. Ital. J. Pediatr..

[36] Lugli L (2021). Neuroprem 2: An Italian study of neurodevelopmental outcomes of very low birth weight infants. Front. Pediatr..

[37] Ancel P-Y (2015). Survival and morbidity of preterm children born at 22 through 34 Weeks’ gestation in France in 2011: Results of the EPIPAGE-2 cohort study. JAMA Pediatr..

[38] Valenzuela-Stutman D. *et al*. & Neocosur Neonatal. Bronchopulmonary dysplasia: risk prediction models for very-low- birth-weight infants. *J. Perinatol.* **39**, 1275–1281 (2019). 10.1038/s41372-019-0430-x 31337853

[39] Fernandez, R., D’Apremont, I., Domínguez, A. & Tapia, J. Red Neonatal Neocosur. Survival and morbidity of very low birth weight infants in a South American Neonatal Network. *Arch. Argent. Pediatr*. **112**, (2014). 10.5546/aap.2014.eng.405 25192520

