# Supplementary material for: Development of a new scoring method in the neurofunctional assessment of preterm infants
Source: Sci Rep. 2022 Sep 29;12:16335. doi: 10.1038/s41598-022-20754-y (PMC9522729; doi:10.1038/s41598-022-20754-y)
Supplement: Supplementary file 1 — Supplementary Information. [file 41598_2022_20754_MOESM1_ESM.docx]

**APPENDIX 1.**

Neurofunctional Assessment of preterm infant at 3 months corrected age

| **ASSESSMENT DOMAINS** | **ITEMS** | | | | **Score** | **CLASSIFICATION** |
| --- | --- | --- | --- | --- | --- | --- |
| 1. REGULATION & ADAPTEDNESS | | State Regulation - Interaction Stability | | |  | 0. typical result: neurofunctional complete patterns    1. slight anomalies which normalize during the exam  2. evident abnormal result: the anomalies remain during examination: the function (adaptive, sensorial, behavioural, motor, adaptive) is moderately troubled, but possible  3. the function is difficult: the anomalies significantly disturb the function  4. severe pathological patterns of movement with anomalies that upset or prevent the function  **OVERALL SCORE (OS):…………………….**  **COMPLEXITY SCORE (CS): ..…………………..**  Diagnosis  …………………………………………………………………………………................................... |
|  |  | Sucking - Feeding Function | | |  |  |
|  |  | Respiratory Pattern and Function | | |  |  |
|  |  | Autonomic – Autonomy | | |  |  |
| 1. NEUROSENSORY FUNCTION | | Fix and Track – Visual Function | | |  |  |
|  |  | Response to Voice - Vocalization | | |  |  |
| 1. BEHAVIOURAL FUNCTION | | Smile - Social Interaction | | |  |  |
|  |  | Excitability – Consolability | | |  |  |
| 1. SPONTANEOUS   MOTOR REPERTOIRE | | Limb Swinging and Wiggling | | |  |  |
|  |  | Kicking | | |  |  |
|  |  | Hand to Hand Contact | | |  |  |
|  |  | Leg Lifting and Foot to Foot Contact | | |  |  |
|  |  | Trunk Rotation | | |  |  |
|  |  | Selective Movements | | |  |  |
|  |  | Hand Regard | | |  |  |
| 1. EVOKED   MOTOR REPERTOIRE | | Postural Patterns | Ventral Suspension  Dorsal Suspension  Lateral Suspension  Axillar Suspension | |  |  |
|  |  | Temporal Patterns | Balancing  Squatting  Lateral Incurvation  Hip Lateral Abduction Reaction | |  |  |
|  |  | Sequences | Axial Rotation  Rolling  Creeping  Rightings | |  |  |
| 1. ACCESSORY NEUROBEHAVIOURAL FACILITATORS | | Facilitation | | Hand Opening  Hip Abduction |  |  |

ASSESSMENT DOMAINS ITEMS

1. **Regulation & Adaptedness**

this area describes the state regulation and stability. It also allows a judgment on the maturation and characteristics of the orogastrointestinal, respiratory and autonomic adaptive functions according to the grading defined above

1. **Neurosensory Function**

this area describes the ability in orientation to visual and auditory stimuli according to the grading defined above

1. **Behavioural Function**

this area describes interactive and socioemotional skills, as well as the features of the action patterns for each age according to the grading defined above

1. **Spontaneous Motor Repertoire**

this area describes the proximal and distal movements and postural patterns of the child are evaluated in detail according to the grading defined above

1. **Evoked Motor Repertoire**

this area describes the motor adjustments during antigravity tests (suspensions), postural adaptability and maintenance during imbalances (temporal models) and the reciprocal relationships between the axial, proximal and distal segments during the execution of the maneuvers (sequences or concatenations) based on the grading defined above

1. **Accessory Neurobehavioural Facilitators items**

this area describes the interventions that facilitate the emergent maturational patterns of the upper limbs (e.g. opening of the hands) and of the lower limbs (e.g. abduction of the hips) according to the grading defined above
